# COVID-19 therapy and prevention

**DOI:** 10.15190/d.2020.10

**Published:** 2020-09-10

**Authors:** Elie Claude-Rosny

**Affiliations:** ^1^Department of Experimental Sciences, Université de Saint-Boniface, Winnipeg, Canada

**Keywords:** Vaccines, outbreaks, coronavirus, SARS-CoV-2 remdesivir, favipiravir, dexamethasone, tocilizumab, hydroxychloroquine, interferon.

## Abstract

Since the outbreak of the new coronavirus pneumonia (COVID-19) in December 2019, more than 23 million people worldwide have been diagnosed with SARS-CoV-2. In response to this pandemic, a global mobilization of scientific, industrial and political support has ensued. However, more than 8 months later, as studies multiply and several governments are embarking on a resumption of their activities, the threat still remains. Our efforts to understand the evolution of the virus and the means to defeat it, at the dawn of a possible new wave, have raised more questions than provided clear and unequivocal answers. Compared to diseases caused by previously known human coronavirus, COVID-19 shows higher transmissibility, as a matter of fact “deeply concerning” cases continue to increase. Under these circumstances, and based on the information we have collected so far, this paper provides an overview of the epidemiological status of COVID-19 by considering, first through comparisons with other coronaviruses, similarities that may guide prevention measures and potentially effective therapies. From this starting point, we aimed to discuss the evidence around the efficacy of masks and respirators for different group of the population. Finally, we address therapeutic aspects including perspectives of vaccines and some antimicrobial agents such as remdesivir, favipiravir, chloroquine, hydroxychloroquine in combination with azithromycin and immunomodulators.

## SUMMARY

*1. Introduction*

*2. Virology and comparison of SARS-CoV-2 with SARS/MERS-CoV*

*3.*** *Incubation, airborne transmissibility and mutation*

*4. Respiratory protection*

*5. Clinical features of COVID-19 and treatment strategies*

- *5.1. Current vaccine studies*

- *5.2. Remdesivir*

- *5.3. Favipiravir*

- *5.4. Immunomodulators*

- *5.5. Dexamethasone*

- *5.6. Chloroquine and its derivatives*

- *5.7. Azithromycin/Hydroxychloroquine*

*6. Conclusion*

## 1. Introduction

**Figure 1:**
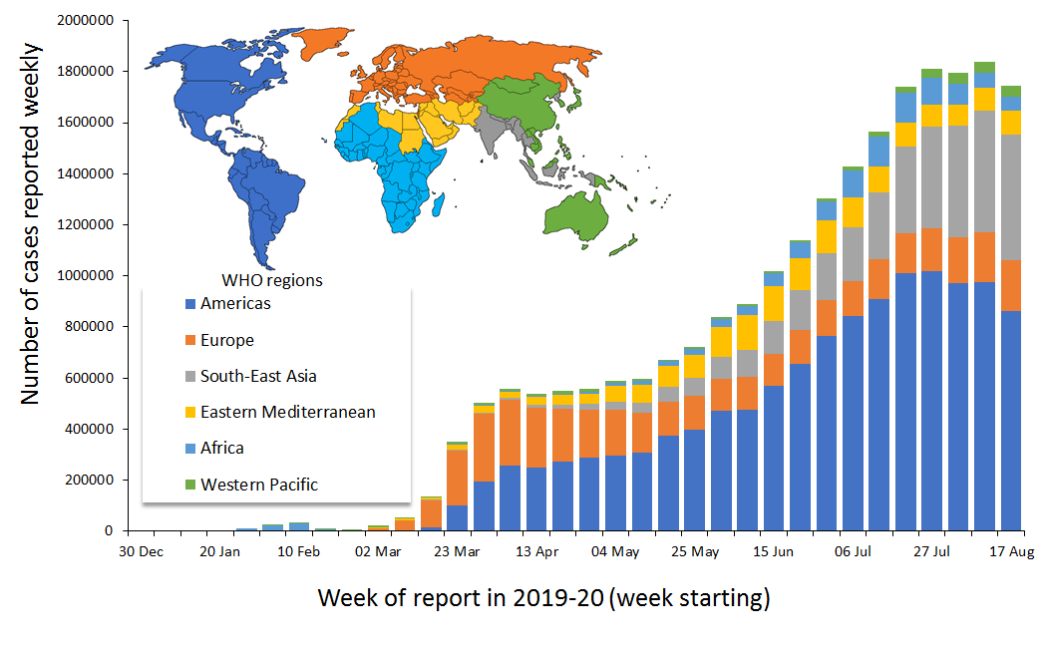
Number of confirmed COVID-19 cases, by date of report and WHO region, December 30, 2019 through August 23, 2020 Adapted from WHO5, under the Creative Commons Attribution 3.0 IGO license (CC BY 3.0 IGO) http://creativecommons.org/licenses/by/3.0/igo/legalcode

The new HCoV was discovered in December 2019 in the city of Wuhan, located in Hubei Province, China^1,2^. Initially informally called "Wuhan Coronavirus", the strain was officially named SARS-CoV-2 by the ICTV on 11 February 2020, based on its taxonomic and phylogenetic similarities with SARS-CoV, which caused the SARS epidemic from 2002 to 2003^1^. The World Health Organization named the disease caused by SARS-CoV-2 coronavirus disease (COVID-19)^1^. The first cases of SARS-CoV-2 were detected in Wuhan, China, while the pattern of human-to-human transmission of the virus was confirmed after it was observed that several individuals who had not visited the city of Wuhan were also affected^3^. The situation rapidly spread beyond the borders of Hubei and China, causing an epidemic of more than 118,000 cases in 114 countries and the subsequent declaration of a pandemic status by the World Health Organization on March 11, 2020^4^. As of August 23, 2020, the number of confirmed cases was 23 077 756, including 800 909 deaths, suggesting a general mortality rate lower than 4%, among confirmed cases, since many infections are asymptomatic or produce mild symptoms, thus, not being diagnosed (Figure 1)^5^. Individuals working in the healthcare sector remain, to this day, among the most affected subjects, as already reported in an ICN report in early May, to the effect that COVID-19 would have infected at least 90 000 healthcare workers. These figures include statistics from only 30 countries^6^.

These mortality rates and the groups of subjects at risk are strongly influenced by many factors, including health prevention measures, individual protection and the extent of early detection of infection, in order to isolate problem cases. The novelty of the disease has caused these issues to be the subject of much debate and even contradiction, such as the capability of the different tests to detect the entire virus rather than debris; arising the concern that such tests are being misinterpreted to suggest COVID-19-positive patients when, in reality, they are not^7,8^. By taking on a dimension where the health and epidemiological stakes are mixed with the political and economic challenges, the situation is sometimes confusing for the layman. This article, therefore, seeks to address the problem of the pandemic by firstly looking at the structural aspect of the virus, secondly at its mode of transmission and life cycle, and finally at some therapeutic and protective approaches developed to combat previous viruses of the human coronavirus family, such as SARS/MERS-CoV.

## 2. Virology and comparison of SARS-CoV-2 with SARS/MERS-CoV

Each SARS-CoV-2 virion has an estimated size of 50-200 nm in diameter, and its shape varies from round to oval^9,10^. The SARS-CoV viral particles, on the other hand, have a smaller diameter of 50-80 nm as measured by electron microscopy^11-13^. The genome of SARS-CoV-2, initially considered stable, consists of a single-stranded RNA of 29,903 nucleotides, containing 14 open reading frames (ORFs), from which four ORFs encode known structural proteins such as S (spike), E (envelope), M (membrane), and N (nucleocapsid) proteins^14,15^. In terms of homology, the genome of SARS-CoV-2 is 50% and 79.5% identical to that of MERS-CoV and SARS-CoV, respectively^16,17^. The similarity between SARS-CoV-2 and SARS-CoV increases to 96%, from a structural point of view, at the level of protein E. This conservation in the E sequence could be explained by its key role as a transmembrane protein^18^. Nevertheless, glycoprotein S, which is responsible for attachment and fusion with the host cell, shows the weakest similarity (76%) between the two specimens SARS-CoV-2 and SARS-CoV^19^. In addition, protein S is believed to have an affinity for the ACE2 receptor that can be exploited in several ways, but the level of comprehension on this matter needs to be further investigated^20-22^. That said, recent studies have failed to demonstrate, beyond doubt, the existence of other virus-like spicule-free structures, despite similar diameter bodies, in autopsies in COVID-19 patients. A cross-species comparison has shown that SARS-CoV-2 is 91% identical to a coronavirus present in Javanese pangolins, with 99% similarity at the site of binding to the ACE2 receptor, ensuring host specificity, and 96% identical to the coronavirus Beta-CoV/bat/Yunnan/RaTG13/2013 found in the Chinese bat, Rhinolophus affinis^23-28^. These similarities leave no doubt as to the origin and the possible crossing of the species barrier.

## 3. Incubation, airborne transmissibility and mutation

**Figure 2:**
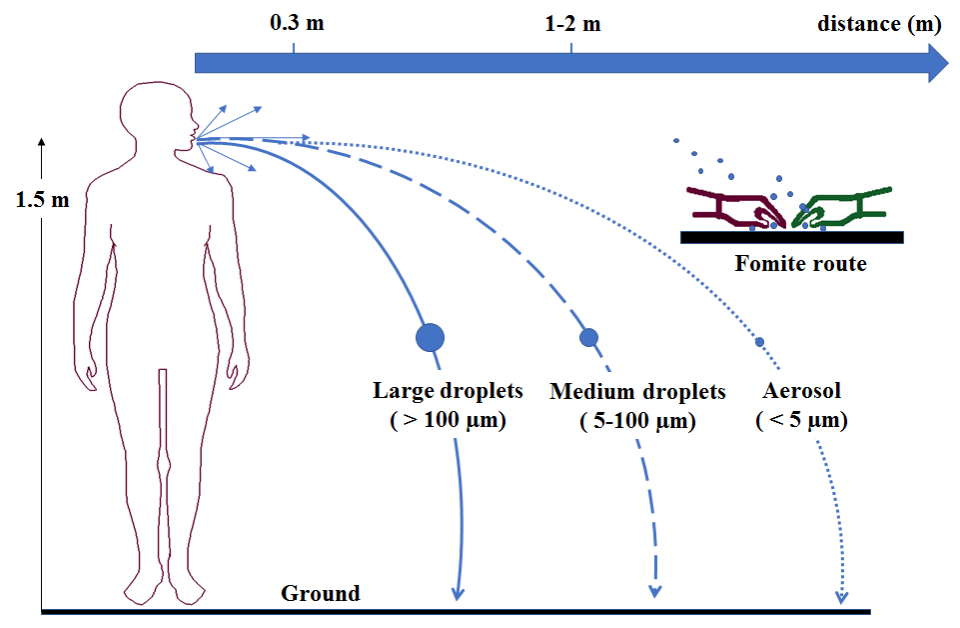
Illustration of different transmission routes of SARS-CoV-2 Aerosols (<5 μm) are responsible for the short-range airborne route, long-range airborne route, and indirect contact route. Large droplets are responsible for the direct spray route and indirect contact route. Fomite route refers to contaminated surfaces. (adapted with permission from references^33,34^)

Once infected, the incubation period of the virus seems, according to the different tests, to be widely variable. The generally advanced interval is 2-14 days^29^. These conclusions are mainly based on 1) WHO observations of 2-10 days; 2) reports from the Chinese National Health Commission initially indicating a period of 10-14 days; and 3) the Centers for Disease Control and Prevention in the USA, which has estimated the incubation period to be between 2 and 14 days^29^. However, this does not exclude longer periods, as reported by Bai *et al.* of 0-24 days^30^. Human-to-human transmission of these types of viruses can occur through direct, indirect (fomite) or respiratory droplet contact (Figure 2). In this respect, a distinction is made between three categories: large droplets (or postilion) which fall to the ground rapidly near the source of emission, medium droplets (or coarse aerosols) with an aerodynamic diameter greater than 5 μm and aerosols with an aerodynamic diameter less than 5 μm^31,32^. Accurate data on how far a droplet or smaller particle and aerosol, emitted by an infected patient, can travel before settling on surfaces are scarce, but it is well known that the distance may vary depending on ventilation, turbulence of the surrounding air, the nasopharyngeal load, and the size of the particle.

While the spread of the virus in the population depends on its transmissibility, R is used as a "diagnostic" tool for the latter. In epidemiology, the basic reproductive number (R) is defined as the expected number of cases of infection directly generated by a case in a population where all individuals are susceptible to the pathogen^35^. For example, measles, which is transmitted by air, has an R between 12 and 18^36^. However, SARS-CoV-2 has an R of about 3, which is far lower than measles, but higher than MERS-CoV and SARS-CoV^37^. On the other hand, its fatality rate would be lower than 4%, as mentioned above, compared to a rate of approximately 10% for measles in some regions, equal to SARS, and 34% for MERS^38^. In sum, as is the trend generally observed for many viruses, low pathogenicity is often associated with high transmissibility, which according to our best observations, appears to be the case for coronaviruses family. This observation seems to become clearer as time progresses. As a matter of fact, in a recent genomic study carried out on the geographical distribution of SARS-CoV-2 mutations, from a library of 48 635 complete genomes, a global emergence of the D614G mutation was observed^39^. This mutation of the SARS-CoV-2 (D614G) protein S is associated with an increase in the number of binding and recognition elements on the surface of the virus for the ACE2 receptor and, consequently, in the capacity of SARS-CoV-2 to infect and transmit to a host. While such a mutation does not seem to be associated with an increase in the pathogenicity of the virus^40^, the fact remains that the D614G strain may not be the only explanation for the very recent change in the profile of the epidemic in the population. Several other hypothetical factors can be considered, such as the average age of newly infected subjects, the widespread awareness of basic sanitary and hygienic measures and the advances, still in progress, of screening methods and care to avoid patients being overlooked. The analysis of this statistical data requires nonetheless great caution in terms of interpretation, since when calculating the fatal risk of infection (IFR), a much lower mortality risk of barely 0.3-0.6% is observed for SARS-CoV-2^41^. Without neglecting the fact that, as we are still in the midst of a pandemic, lack of hindsight, the results are still fragmentary for the moment. Let us also add that it is difficult to clearly detect and prevent transmission in a population, except through extensive screening, due in particular, to a transmission rate by asymptomatic (or mildly symptomatic) subjects of about 55%^42^. For these reasons, concerning asymptomatic cases that are difficult to assess, the effects and transmissibility of SARS-CoV-2 between mother and foetus are all the more still poorly known. The same is true for transmission via breast milk^43^.

## 4. Respiratory protection

Before discussing treatments, let us first address the issue of respiratory protection. As mentioned above, SARS-CoV-2 is mainly spread through the airways via fine aerosol droplets, although the definition of aerosol itself is still debated. Significant loads of SARS-CoV-2 have been reported in the saliva of patients with COVID-19, not to mention growing voices calling for recognition of the potential for airborne spread of the virus^44-46^. As a result, several studies have investigated the effectiveness of various nasobuccal means of filtering these transmission vectors. Currently, three types of respiratory protection are mainly suggested and put forward in the literature and by certain health authorities. These are respirators, medical masks and homemade masks. In a meta-analysis of 19 randomised clinical trials, conducted by MacIntyre and Chughtai, on the use of respiratory protection by healthcare workers, sick patients and individuals randomly selected from the population, the findings suggest a benefit of wearing a mask superior to that associated with hand hygiene alone (results were here reported according to the PRISMA criteria)^47^. In the population, in community settings with conditions highly favourable to transmissions, such as households or colleges, the practice of mask use and hand hygiene together would further increase the protective effect. Randomised controlled trials among healthcare workers indicate that respirators, when worn throughout a shift, are effective, but not when worn intermittently. Medical masks are not sufficiently effective for this group of workers, while homemade tissue masks are even less effective^47^. Moreover, it appears that the effectiveness of homemade masks is strongly linked to several factors, such as the nature of the fabric, the design and their washing capacity, even suggesting, but without generalising, a risk of increased infections caused by the homemade masks themselves. In short, homemade masks are not a recommended option for healthcare workers^47^. None of these trials, however, have examined the combined or unique effect of visors alone or in conjunction with other protective equipment or practice in the population.

## 5. Clinical features of COVID-19 and treatment strategies

To date, to the best of our knowledge, there is no effective vaccine developed or recognised by the WHO against SARS-CoV-2. However, in order to better synthesize such treatment, different animal models have been developed to study the pathogenicity of the virus on organs, similar to what we could observe in clinical cases in humans. These include hACE2 transgenic mice in which symptoms of pneumonia were reproduced with SARS-CoV-2; that is, weight loss and intestinal pneumonia comparable to the initial clinical reports of pneumonia observed in COVID-19^48^. The main clinical signs of the disease in humans, listed in Table 1, as recently reported in the literature, include fever, cough, dyspnea, dysgeusia and hyposmia^49^. It is obviously not possible to diagnose COVID-19 on the basis of these symptoms alone, but they should, when they occur, lead to a serious examination of the condition of the potentially infected patient.

**Table 1:** Symptoms of COVID-19 from British Medical Journal^49^

Factor	Prevalence (%)		Characteristics of symptoms	
		Key diagnostic	Common	Uncommon
Fever	78	X	X	
Cough	57	X	X	
Dyspnea	31-40	X	X	
Dysgeusia/Hyposmia	38 / 41	X	X	
Fatigue	31		X	
Arthralgia or Myalgia	11-17		X	
Expectoration	23.7		X	
Chest tightness	22.9		X	
Sore throat	12		X	
Gastrointestinal.symptoms	20			X

### 5.1 Current vaccine studies

In this ongoing effort to understand the disease and develop a vaccine against it, as of August 2020, the global COVID-19 vaccine research and development landscape includes a large number of candidates, of which many are currently at exploratory or preclinical stages. Table 2 lists some of the most actual advanced projects in the clinical development phases, although other initiatives exploiting innovative technology platforms are continuously announced by academic and industrial institutions. The technology landscape presented in Table 2 is intended to illustrate, while inviting the reader not to be limited to it, the variety of potential targets that can be exploited.

**Table 2:** Ongoing clinical trials for potential vaccines against Sars-CoV-2

Vaccine prospect	Developer	Technology		Evolution of trial	
			Phase	Number of Participants	Duration (MM/YY)
NVX-CoV2373 ^50^	Novavax	recombinant spike protein	I	131	05/20 - 07/21
SCB-2019 ^51^	Clover Biopharm	spike protein trimeric subunit	I	150	06/20 - 03/21
AG0301-COVID19 ^52^	AnGes Inc.	DNA plasmid	I - II	30	06/20 - 07/21
Ad5-nCoV ^53^	CanSinoBiologics	recombinant adenovirus vector	II	382	03/20 - 12/20
AZD1222 ^54^	Univ. Of Oxford	adenovirus vector	II - III	10 260	05/20 - 08/21
BNT162 ^55^	BioNTech	RNA	II - III	30 000	04/20 - 05/21
CoronaVac ^56^	Sinovac Biotech	inactivated SARS-CoV-2 virus	III	10 490	04/20 - 12/20
BBIBP-CorV ^57^	Sinopharm	inactivated SARS-CoV-2 virus	III	15 000	07/20 - 07/21
mRNA-1273 ^58^	Moderna	nanoparticle	III	30 000	07/20 - 10/21

**Figure 3:**
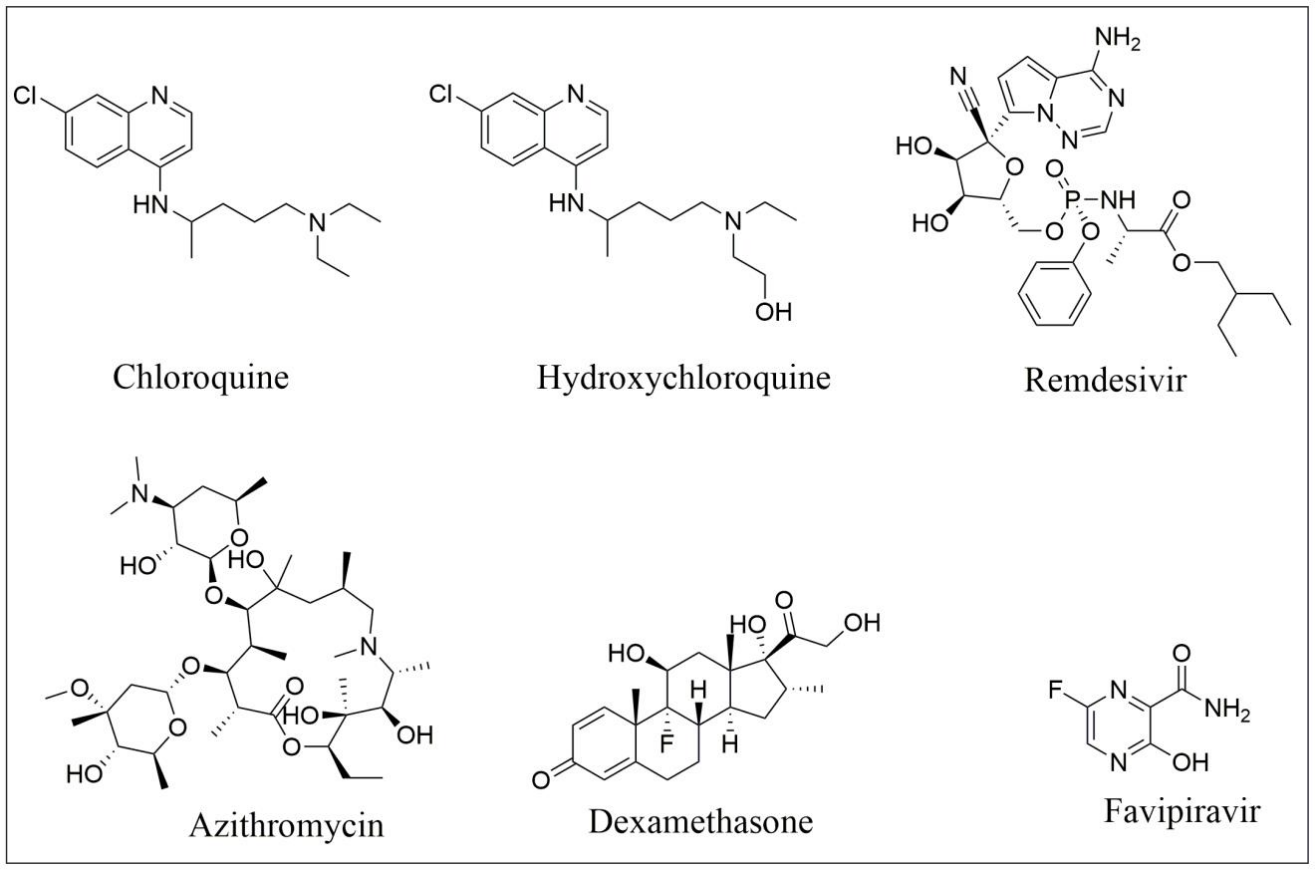
Structures of antimalarials chloroquine and hydroxychloroquine, antivirals remdesivir and favipiravir, corticosteroid dexamethasone and antibiotic azithromycin

Nonetheless, the discovery of a vaccine is not the only possible and feasible avenue we should emphasise for prevention and treatment of the disease. Molecules capable of directly attacking the virus are being tested (Figure 3). That said, despite more than 200 clinical trials, no curative compound has been proven, by broad consensus, to be effective against SARS-CoV-2. Studies at this level have focused primarily on antivirals, antibiotics combinations and immunomodulators. Among the many proposals in the literature, our review has focused on approaches that have appeared to achieve a degree of agreement and that would allow access to the highest possible number of individuals, taking into account production costs, availability and the therapeutic profile.

### 5.2 Remdesivir

A monophosphate derivative of an adenine nucleoside analogue, remdesivir was initially developed to treat Ebola virus disease and Marburg virus infections^2,59^. Its antiviral action targets a wide range of RNA viruses, including potentially SARS/MERS-CoV, thus presumably SARS-CoV-2. As such, Wang *et al.* have reported an inhibitory action of remdesivir on human Huh-7 cancerous liver cells known to be susceptible to SARS-CoV-2^60^. In addition, more recently, Pruijssers *et al.* demonstrated strong inhibition of SARS-CoV-2 replication in cultures of human lung cells and primary human airway epithelium in the presence of remdesivir (EC50= 0.01 µM)^61^. This is consistent with the ability of these cells to metabolize the prodrug, in contrast to infected Vero E6 cells, which have a significantly lower sensitivity to remdesivir (EC50= 1.65 µM). In vivo results in mice infected with a chimeric SARS-CoV virus also demonstrated an improvement in lung function, accompanied by a decrease in viral load^61^. Other as yet preliminary studies also address this promising avenue, indicating a reduction in hospitalization time, with remdesivir^62^.

### 5.3 Favipiravir

Favipiravir, or T-705, is an organofluorinated pyrazine that is believed to act by selective inhibition of RNA-dependent RNA polymerase from RNA viruses (RdRp), since it does not interact with DNA transcription to RNA or DNA replication in mammalian cells^63^. Approved since 2014, in Japan, to treat influenza strains that cause more severe disease and resistant to existing antiviruses, its action has been studied in February 2020 against SARS-CoV-2^64,65^. It appears to be more effective than the lopinavir/ritonavir combination according to a preliminary study carried out on 80 patients^66^.

### 5.4 Immunomodulators

The inflammatory and immune dimension of COVID-19 is another aspect that one can address, through the beneficial effects of interleukin inhibitors and anti-inflammatory drugs. Increasing evidence have suggested a correlation between the SARS-CoV-2 infection and interleukin-6 (IL-6) production^67-70^. This massive induction of interleukin, generating a "cytokine storm", is believed to be responsible for the severe inflammatory pulmonary response in infected patients. The interest of interleukin inhibitors is, therefore, to reduce this pulmonary inflammatory response. A very early study has demonstrated the 45% reducing effect of tocilizumab on the risk of mortality compared to a control group receiving only respiratory assistance. However, tocilizumab was associated in patients in this study (n=78) with an increased incidence of superinfections (54% vs 26%; P<0.001), although this does not appear to significantly affect the long-term fatality rate between tocilizumab-treated patients with superinfection versus those without superinfection (22% vs 15%; P=0.42)^71^. Interestingly, another IL-6 inhibitor, sarilumab, have not shown notable benefit on clinical outcomes in the preliminary phase II analysis, conducted by Sanofi and Regeneron, when comparing "severe + critical" groups versus placebo. The discrepancy between these two treatments, in an effort to treat COVID-19, is not well understood or explained in the literature at this time and more evidence remains to be generated.

Besides, in light of the current knowledge of the beneficial effects of both the immunomodulators and the antiviral remdesivir discussed earlier, a randomized controlled clinical trial was initiated to evaluate the safety of a regimen of remdesivir with the immunomodulator interferon beta-1a in patients with COVID-19^72^. One of the reasons for this initiative is that, at present, an improvement in median recovery time of approximately 4 days is observed in subjects receiving remdesivir with a mortality rate of 7.1% versus 11.9% for the placebo group^62,73^. In order to significantly improve this mortality rate, a research run by the U.S. National Institute of Allergy and Infectious Diseases (NIAID) has therefore proposed a combination with other therapeutic agents^73^. In addition, the inhibitory effect of interferon type 1 was observed in the laboratory on the SARS-CoV, SARS-CoV-2 and MERS-CoV coronaviruses^72^. As the combination of remdesivir and interferon (beta-1a) has never been evaluated in a large randomized trial, this information opened the door to a probable new treatment alternative. As a result, the Adaptive COVID-19 Treatment Trial 3 (ACTT 3), sponsored by NIAID and which began on February 21, 2020, plans to recruit 1,000 adults with COVID-19. The goal of this controlled clinical trial is to study, in its methodology, the effect of remdesivir plus interferon beta-1a versus remdesivir alone on the duration of hospitalization and ultimately mortality of COVID-19 subjects. Preliminary results will be available by fall 2020^72^.

### 5.5 Dexamethasone

In the same spirit of studying the consequences of "cytokine storm", it has been observed that the severe form of COVID-19 is associated with interstitial pneumonia and then with alveolar damage that can precipitate acute respiratory distress syndrome (ARDS)^74^. Due to their anti-inflammatory and immunomodulatory properties, inducing the activation of the endothelial nitric oxide synthase (eNOS)^75^, corticosteroids have been the subject of studies evaluating their ability to reduce systemic and pulmonary damage in patients with an ARDS. Therefore, dexamethasone presented itself as a suitable candidate. Indeed, it is currently recommended for many conditions such as inflammatory problems, dermatological affections, allergies, respiratory diseases etc. Horby *et al.* published the preliminary results of a treatment arm of the national open-label study Recovery which is evaluating the efficacy and safety of several potential treatments of COVID-19^76^. Among the possible treatment proposals, subjects treated with dexamethasone had a statistically significantly lower incidence of mortality compared to the standard care group receiving invasive mechanical ventilation (29.3% vs. 41.4%; ratio, 0.64; 95% CI, 0.51-0.81)^76^. The effect was less marked compared to subjects under oxygenation without invasive mechanical ventilation (23.3% vs. 26.2%; ratio, 0.82; 95% CI, 0.72-0.94), whereas no significant effect was observed in patients not requiring any oxygenation support (17.8% vs. 14.0%; ratio, 1.19; 95% CI, 0.91-1.55)^76^. In addition, dexamethasone provides this major element in that, although remdesivir may shorten the recovery time in hospitalised patients, no therapeutic agent has so far been shown to be capable of reducing significantly and unequivocally mortality like dexamethasone. That being said, it should be borne in mind that, by its very nature, dexamethasone has typical side effects associated with corticosteroids, in addition to being recommendable, depending on the situation, only at the severe stage of the disease, as defined by the Infectious Diseases Society of America (IDSA)^77^.

### 5.6 Chloroquine and its derivatives

In this category, chloroquine is among the first molecules to have aroused the interest of researchers. Commonly used as an affordable antimalarial and immunosuppressant, chloroquine is believed to act by alkalinising the endosomal pH required for virus infection while interfering with the glycosylation of the cellular receptors for SARS-CoV^78^. Chloroquine would presumably be effective against the SARS-CoV-2 virus at EC50 concentrations of 1.13 µM associated with CC50 toxicity at concentrations of 100 µM, or almost two orders of magnitude higher^79^. Its use, however, also remains off-label for potential treatment of the disease^80^. Moreover, according to several authors, it is important to remain cautious and closely monitor the condition of patients receiving chloroquine^2,81^. In order to reduce the risks associated with the side effects to be monitored, other alternatives to chloroquine have been proposed, such as hydroxychloroquine, which is a derivative of chloroquine bearing an hydroxyl group (Figure 3)^82^.

### 5.7 Azithromycin/Hydroxychloroquine combination

Although it may seem counterintuitive to use antibiotics in the treatment of a viral disease, we have long known that the risk of bacterial superinfection plays a role in the severity of the influenza virus infection^83^. It is therefore not uncommon to combine antibiotics with antivirals in order to improve the synergistic effect and reduce the prognosis of complications. Thus, based on proposals submitted by recent papers reporting an inhibitor effect of chloroquine on the growth of SARS-CoV-2 *in vitro*^60^ and *in vivo*^79^, Raoult *et al.* reported in March 2020 that the combination of hydroxychloroquine (HCQ) and azithromycin allowed them to treat COVID-19^84^. As mentioned above for its analogue, the chloroquine, precautions should be taken with hydroxychloroquine regarding its use and monitoring of the patient's cardiac, hepatic and renal condition is recommended. Although the risks remain low at the recommended daily doses (< 1000 mg) of HCQ^85,86^. This notwithstanding, based on the balance of risks versus benefits, the use of chloroquine and hydroxychloroquine has been revoked by the FDA, as part of the emergency use authorisation (EUA), to treat certain patients hospitalised with COVID-19 when no clinical trial is in place^87^. According to the agency, the legal criteria for decreeing such an emergency authorisation are no longer met. A decision by the American College of Physicians is also in the same vein regarding the use of hydroxychloroquine, alone or in combination with azithromycin, as a preventative or for the treatment of coronavirus disease^88^.

## 6. Conclusion

This article reviews several published prevention and treatment approaches against the new coronavirus. The SARS-CoV-2 virus has demonstrated that it can be fatal, especially for the patients belonging to one of the risk groups, with a transmission potential that should not be overlooked. To date, more than 23 million confirmed cases and 800 909 deaths have been recorded worldwide, hence the importance of taking this pandemic seriously and focusing efforts to find a cure and prevent its spreading. By first comparing SARS-CoV and MERS-CoV, it is possible to establish similarities that can guide potentially effective therapies. That said, no vaccine or antivirus has yet been recognised and supported by conclusive data as being fully effective against SARS-CoV-2. Already known compounds, such as anti-inflammatory steroid dexamethasone and the antiviral medication remdesivir, either in combination or alone, appear to be a fast and appealing route of treatment as several encouraging results suggest that COVID-19 can possibly be treated with the medical arsenal we already have at our disposal.
